# Malassezia Folliculitis: Pathogenesis and Diagnostic Challenges

**DOI:** 10.7759/cureus.73429

**Published:** 2024-11-11

**Authors:** Jesús Iván Martínez-Ortega, Jacqueline E Mut Quej, Samantha Franco González

**Affiliations:** 1 Dermatology, Dermatology Institute of Jalisco, Zapopan, MEX; 2 Histology, Autonomous University of Nuevo Leon, Faculty of Medicine, Monterrey, MEX; 3 Internal Medicine, Regional General Hospital No. 12 IMSS Lic. Benito Juárez, Merida, MEX; 4 Internal Medicine, National Medical Center 21st Century, Ciudad de México, MEX

**Keywords:** fungal infections, fungal skin disorders, hydrophobic interactions, immune-mediated fungal infections, immunopathogenesis, keratinocyte response, lipophilic fungi, malassezia folliculitis, pityrosporum folliculitis, yeast infections

## Abstract

*Malassezia *folliculitis (MF) is a fungal infection that often presents with pruritic follicular papules and pustules, primarily affecting the upper body. Due to its clinical similarity to bacterial folliculitis, misdiagnosis and delayed treatment are common. In this report, we present the case of a 16-year-old male who developed persistent pruritic papules on his upper back and chest, initially misdiagnosed as bacterial folliculitis. Following ineffective antibiotic treatment, mycological analysis confirmed *Malassezia* as the causative pathogen. This case emphasizes the importance of considering fungal etiologies in folliculitis, particularly in patients with recurrent or treatment-resistant symptoms. The patient’s condition improved significantly following antifungal therapy, underscoring the need for accurate diagnosis and appropriate treatment.

## Introduction

Folliculitis is an inflammatory condition that affects the superficial or deep portions of hair follicles and can result from a variety of etiologic factors, including bacterial, viral, and fungal infections, as well as non-infectious causes such as drug reactions or eosinophilic infiltration. One notable infectious cause is *Malassezia *spp., a genus of lipophilic yeast that is part of the normal skin flora but can become pathogenic under certain conditions. When this yeast causes folliculitis, it leads to a specific form known as *Malassezia *folliculitis (MF), formerly known as *Pityrosporum* folliculitis. Clinically, MF is characterized by dome-shaped papules, pustules, and, in more severe cases, nodules or cysts, often accompanied by pruritus. The lesions tend to primarily affect sebaceous-rich areas such as the chest, back, and shoulders, though they can also appear on the neck and upper limbs [1,2].

Managing MF poses a clinical challenge due to its similarity to other forms of folliculitis, particularly bacterial folliculitis and acneiform eruptions. This overlap often results in misdiagnosis and the inappropriate use of antibiotics or acne medications, which may exacerbate the condition. Correctly distinguishing MF from other forms of folliculitis is crucial to initiating appropriate antifungal therapy [2].

## Case presentation

A 16-year-old male from Mérida, Yucatán, presented with multiple acneiform lesions that had persisted for three months. He had no relevant medical history, and no prior chronic conditions or allergies were reported. The patient initially received two intramuscular injections of dexamethasone (8 mg/2 ml, one injection every 72 hours) and topical mupirocin, applied twice daily for one week. However, these treatments did not lead to any improvement; in fact, the lesions seemed to worsen. There were no systemic symptoms such as fever, malaise, or weight loss during the condition.

**Figure 1:**
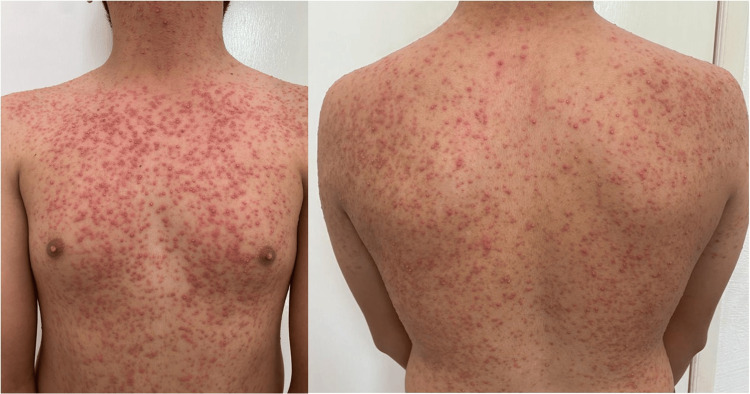
Numerous pustules on the neck, as well as the anterior and posterior trunk.

A disseminated dermatosis was observed on physical examination, affecting the neck, trunk, and upper limbs. The lesions were characterized by multiple follicular papulopustular eruptions (Figure 1). The unusual distribution, monomorphic appearance, and absence of typical facial acneiform lesions and comedones prompted further investigation.

**Figure 2:**
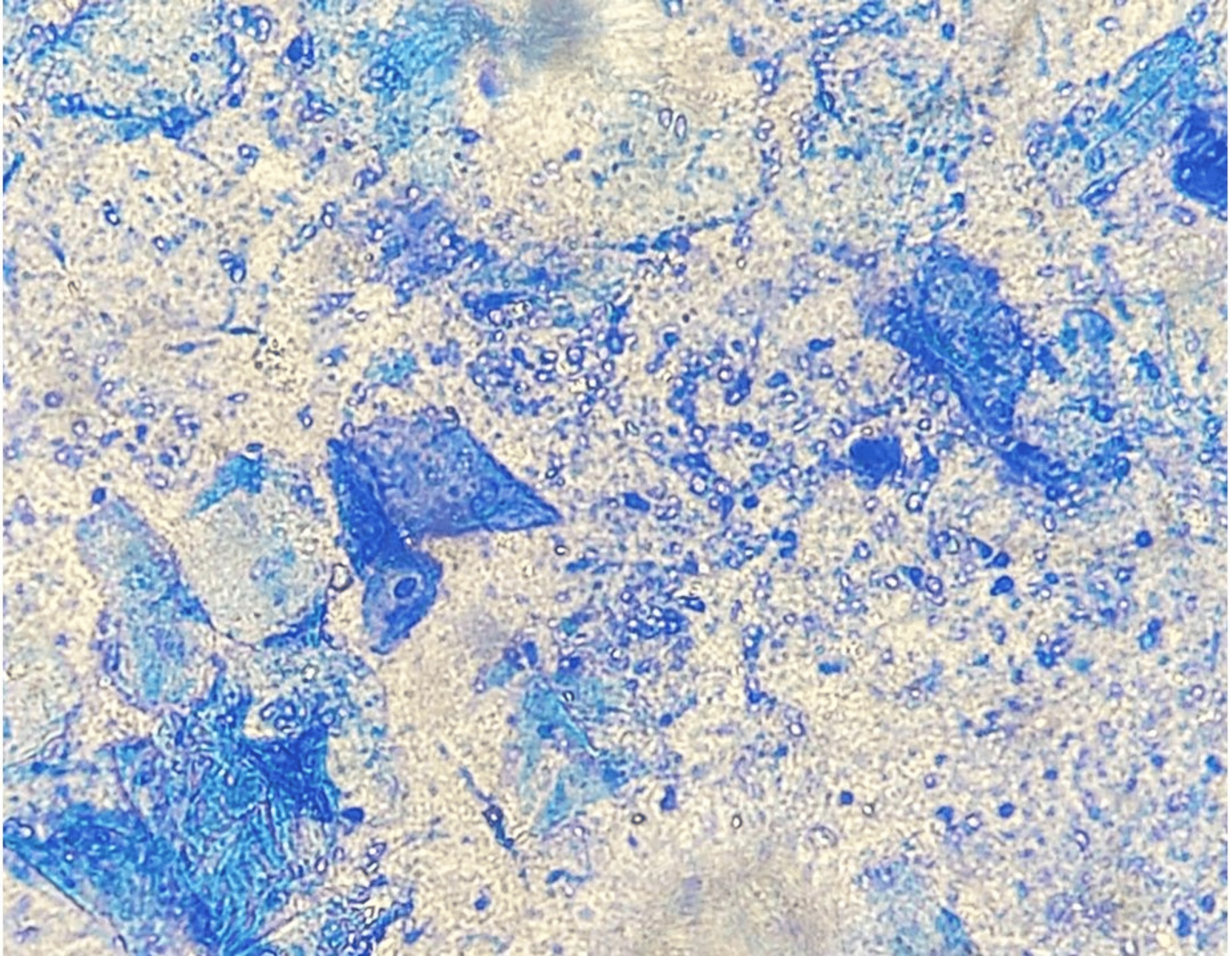
Direct microscopic examination of pustule fluid stained with methylene blue (40x), revealing clusters of yeast consistent with Malassezia spp.

Laboratory studies, including complete blood count, liver and renal function tests. To further investigate the etiology, a direct examination of the fluid from some pustules was performed. The sample was stained with methylene blue on a microscope slide, revealing abundant yeast cells consistent with *Malassezia *spp. (Figure 2). Based on these findings, the patient was diagnosed with MF.

Treatment was initiated with oral itraconazole 100 mg daily for one month, along with 2% topical ketoconazole shampoo. Complete resolution was achieved within 30 days, with no recurrence of lesions or pruritus during the three-month follow-up while continuing ketoconazole shampoo three times a week.

## Discussion

These yeasts are the primary eukaryotic commensals on human skin, playing a dominant role in the skin's microbiome. The colonization rate of *Malassezia *spp*. *in humans varies but can reach as high as 98%. Research has identified 18 different species of *Malassezia*, among which *M. furfur, M. globosa, M. restricta, M. sympodialis,* and *M. pachydermatis* have most frequently been isolated from MF lesions [3,4].

MF primarily affects young and middle-aged adults and is rare in children. In adolescents, it often coexists with acne. While comprehensive prevalence data in the general population is lacking, hospital studies report rates ranging from 1% to 2% to as high as 16%, depending on the country [2,4].

Like dermatophytes, commonly reported predisposing factors for MF include excessive sweating, hot climates, physical activity, recent use of antibiotics or corticosteroids, and immunosuppression. However, unlike dermatophyte infections and more similar to *Cutibacterium acnes, *high sebum production may play a more specific role in MF [2,5].

*Malassezia *spp*. *species are lipophilic, thriving in seborrheic areas like the scalp and trunk due to their dependence on external long-chain fatty acids for energy and structural components, as they cannot synthesize these on their own. They exploit skin surface lipids to meet their nutritional needs [2,6].

Recent research has demonstrated that *Cutibacterium acnes*, a common skin commensal, can induce epidermal lipid synthesis. This observation may suggest a possible explanation for the frequent coexistence of MF with acne, as increased lipid availability could hypothetically promote *Malassezia *proliferation in seborrheic areas. However, this connection remains speculative [7]. Beyond causing MF, these fungi are also associated with conditions like seborrheic dermatitis and pityriasis versicolor [2,6]. 

MF is characterized by monomorphic, follicular, dome-shaped papules and pustules, predominantly affecting seborrheic areas such as the upper trunk. Pruritus is common, occurring in more than half of cases. The absence of comedones helps differentiate MF from acne vulgaris, which typically affects both the face and trunk, often without significant itching, and presents with comedones, papules, pustules, and nodules. Other important differential diagnoses include acneiform eruptions, which present similarly to MF with erythematous pustules and papules on the trunk, shoulders, and face, often requiring a good clinical history. Bacterial folliculitis tends to involve deeper portions of the hair follicle, sometimes resulting in purulent discharge or fever. Eosinophilic folliculitis, associated with elevated eosinophil counts, can be linked to hematologic cancers and HIV infections [2,4].

Additionally, the use of steroids may lead to an acneiform eruption that is more monomorphic and acute, typically lacking comedones, compared to typical acne. In such cases, the chronological history and patient interrogation should raise suspicion regarding this diagnosis. Importantly, in the present case, this possibility was ruled out, as the steroids were administered after the onset of the eruption, not before it [8].

Bacterial folliculitis may represent the most challenging differential diagnosis for MF. Similarly, acneiform eruptions can be difficult to differentiate, especially if a detailed history is not available. However, direct examination of the pustule content remains an easy, practical, and effective method for establishing a diagnosis in uncertain cases. Less commonly, conditions such as viral folliculitis, Demodex folliculitis, the acneiform presentation of cutaneous lymphomas, and Gram-negative folliculitis may mimic MF. The differential diagnosis should consider these conditions, especially when typical treatments fail to resolve symptoms [2,4,8,9].

We recommend performing a dermoscopy and Wood's light examination in all cases. If microscopic analysis is unavailable, the recent position statement from the European Academy of Dermatology and Venereology (EADV) suggests relying on clinical assessment alone. Furthermore, given that some studies indicate topical antifungals have a 100% cure rate with mild adverse events, a therapeutic trial lasting two to four weeks aimed at achieving significant improvement rather than complete resolution may be a practical approach in that context [4].

For more complex situations, Table 1 summarizes the available diagnostic tools based on the EADV statement [4], including their pros, cons, and best-use cases.

**Table 1:** Diagnostic methods for Malassezia folliculitis: pros, cons, and best use cases. This table summarizes various diagnostic methods used for detecting *Malassezia *folliculitis (MF). It provides an overview of the advantages, limitations, and ideal clinical scenarios for each technique. Direct examination methods like KOH and Gram staining are practical and widely available, especially in routine and resource-limited settings. More advanced methods like fluorescence microscopy, molecular-based detection, and fungal culture offer higher specificity but are less accessible due to cost and equipment requirements. Histopathological examinations are ideal for complex or treatment-resistant cases, while non-invasive techniques like dermoscopy and Wood’s lamp serve as valuable adjuncts for clinical assessment, though they are not conclusive without laboratory confirmation. The table highlights how these tools can complement each other in the diagnostic workflow for MF. This table is primarily based on the position statement from the European Academy of Dermatology and Venereology (EADV). MALDI-TOF MS: matrix-assisted laser desorption ionization-time of flight mass spectrometry, PCR: polymerase chain reaction, RFLP: restriction fragment length polymorphism.

Method	Pros	Cons	Best use case
Direct exam			
KOH staining	Quick, widely available, detects unipolar budding yeasts. Applied on pustule content for better yield.	Differentiating yeast from bacteria can be challenging for non-experts.	Best for routine clinical settings, especially in resource-limited environments.
Gram staining	High sensitivity and specificity, effective in distinguishing between bacteria (Gram-positive) and yeasts.	Requires more steps and is not as readily available as KOH.	Excellent for distinguishing bacterial and fungal infections. Ideal when co-infection is suspected.
Methylene blue staining	Enhances the visibility of fungal elements, can be applied directly onto the sample.	Does not provide additional cytological detail.	Best for enhancing visibility when needed, especially in direct pustule content.
May–Grünwald–Giemsa (MGG)	Highly sensitive and provides additional cytological details such as inflammatory cells.	Requires more time and resources than KOH. Not widely available in all settings.	Ideal for more complex cases where both fungal and inflammatory components need to be evaluated.
Tape stripping	Non-invasive, effective for superficial infections like Pityriasis versicolor.	Ineffective for MF due to its follicular involvement, which requires sampling of deeper skin layers.	Best suited for superficial fungal infections but not suitable for diagnosing MF.
Dermoscopy	Quick, non-invasive, visualizes folliculocentric papules and pustules, helpful in suspecting MF.	Cannot directly diagnose MF; requires laboratory confirmation.	Best as an adjunct for initial suspicion of MF, useful in clinical assessment but not for final diagnosis.
Fluorescence microscopy (Calcofluor White)	High sensitivity when combined with KOH, good visualization of fungal elements.	Requires specialized equipment, limiting its use in routine clinical settings.	Best used in specialized settings where fluorescence microscopy is available.
Histopathological examination (HE, PAS)	Detects cases missed by direct examination, especially in complex or non-responsive cases. Provides detailed analysis of inflammation and follicular plugging.	Invasive (requires punch biopsy), resource-intensive, not available in all clinics.	Ideal for cases where treatment is failing or for confirming a diagnosis in complex scenarios.
Molecular-based detection (PCR, MALDI-TOF MS)	Highly specific, able to identify *Malassezia* species/strains and support diagnosis. Can identify species and provide genotypic information.	Expensive, not routinely available, and cannot distinguish between pathogenic and normal skin flora. PCR variants (e.g., RFLP, tRFLP) add complexity.	Useful in research settings or when species-specific information is required.
Fungal culture	Can confirm diagnosis and susceptibility to antifungals. Provides detailed information on antimycotic susceptibility.	Risk of contamination by non-pathogenic *Malassezia* from adjacent skin. Slow growth of some species biases results. Rarely performed due to these limitations.	Best for confirming antifungal resistance, but not routinely recommended due to limitations and contamination risk.
Wood’s lamp	Quick, inexpensive, helps rule out associated conditions like Pityriasis versicolor, Erythrasma, or *Microsporum canis* (Tinea).	Limited utility for MF, low sensitivity (65.3%) for MF diagnosis.	Best for ruling out associated conditions, though not directly diagnostic for MF. However, a positive result may support the diagnosis. It's a quick and cost-effective tool.

Treatment with topical azole formulations is generally effective. However, systemic treatment with itraconazole or fluconazole may be necessary in special cases, such as patients with immunosuppression. In our case, the extent of the affected area led us to prefer oral therapy. It's important to note that many patients may experience relapses, and a low-dose maintenance regimen can help manage recurrent episodes. Additionally, incomplete resolution or lack of response may occur, in which case the diagnostic approach should be re-evaluated. As discussed, when *Malassezia *spp*. *is confirmed via direct exam, co-existing conditions like truncal acne are common and may require additional treatment [4,10].

Murine models of epicutaneous infection have demonstrated that *Malassezia *spp. selectively induce interleukin-17 (IL-17) and related cytokines. In a study using fluorescence-activated cell sorting (FACS) of various myeloid skin cell subsets, neutrophils, and Langerhans cells were found to express the highest levels of IL-23 transcripts [6]. More recent studies have broadened our understanding of the immunopathogenic mechanisms, revealing that epicutaneous administration of *Malassezia *spp*. *prompts human keratinocytes to secrete IL-23 through the TLR2/MyD88/NF-κB signaling pathway [11]. This triggers the differentiation of Th17 cells. Additionally, γδ T cells, αβ T cells, and T-cell receptor (TCR)-negative innate lymphoid cells (ILCs) have been shown to express IL-17, which may contribute both to fungal clearance and immunopathogenesis. The latter could explain clinical features, such as epidermal thickening and scaling, in conditions like seborrheic dermatitis [3].

In MF, it is hypothesized that certain paracrine factors may influence neutrophil activation, leading to their accumulation in the pilosebaceous unit and the formation of follicular pustules. We previously proposed that physicochemical forces, specifically the hydrophobic effect, might drive lipophilic fungi [12] or bacteria [13] into proximity, transforming commensal organisms into pathogens. The lipids in the hydrophobic matrix of the stratum corneum, as well as sebaceous gland secretions into the hair follicles, likely create an environment that facilitates the aggregation of hydrophobic fungi. This accumulation may attract neutrophils to the pilosebaceous unit, leading to their localized buildup. This hypothesis might explain why clinical manifestations predominantly occur in seborrheic areas, such as the trunk, as observed in this patient [12,13].

**Figure 3:**
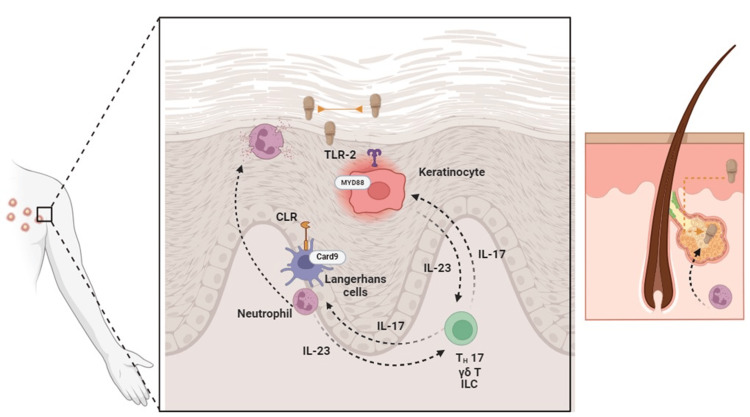
Diagram depicting the immunopathogenesis of the host response to Malassezia yeast. In the upper section, orange arrows represent the proposed hydrophobic effect, which may drive yeast clustering and its approach to other lipidic macromolecules, such as the lipid barrier in the epidermis and sebaceous secretions from the sebaceous glands of the pilosebaceous unit (seen on the right side of the image). Toll-like receptor 2 (TLR-2) on keratinocytes, along with other potential receptors like interleukin-36 receptor (IL-36R), senses the presence of the yeast. This detection triggers a signaling cascade through myeloid differentiation primary response 88 (MYD88), ultimately leading to the production of interleukin-23 (IL-23). Other immune cells, such as neutrophils and Langerhans cells, recognize *Malassezia *through C-type lectin receptors (CLRs) and CARD9, contributing further to IL-23 production. IL-23 plays a key role in the differentiation of T cells into the Th17 profile. Additionally, other immune cells, such as γδ T cells and innate lymphoid cells (ILCs), have been shown to produce interleukin-17 (IL-17). This cytokine not only activates neutrophils to eliminate the yeast but may also be responsible for clinical manifestations, such as the accumulation of polymorphonuclear cells (PMNs) at the sites of *Malassezia* yeast clusters and the formation of pustules. TLR-2: Toll-like receptor 2; IL-36R: interleukin-36 receptor; MYD88: myeloid differentiation primary response 88; IL-23: interleukin-23; CLR: C-type lectin receptors; CARD9: caspase recruitment domain-containing protein 9; Th17: T helper 17 cells; IL-17: interleukin-17; PMN: polymorphonuclear cells. Image created with Biorender.com. Credits to Jesús Iván Martínez Ortega, MD.

As with other fungal infections, such as those caused by dermatophytes [5] or dimorphic fungi [14], the IL-23/IL-17 axis is crucial in mediating the immune response against *Malassezia*. Additional immune cells, including natural killer (NK) cells, may also contribute to IL-17 production. Other receptors, like IL-36R and the aryl hydrocarbon receptor (AhR), may be involved in keratinocyte recognition of *Malassezia *spp., further influencing the immune response [15] (Figure 3).

## Conclusions

*Malassezia *folliculitis (MF) is a common yet often misdiagnosed condition due to its clinical similarity to bacterial folliculitis and acneiform eruptions. Mismanagement, particularly with antibiotics, can exacerbate the condition. Correct diagnosis through clinical assessment and laboratory confirmation is critical to ensure appropriate antifungal treatment. Given the recurrence potential of MF, a thorough understanding of the condition and its differentiation from other causes of folliculitis is necessary for effective, long-term patient management. Future research should focus on optimizing diagnostic tools and exploring new antifungal therapies for resistant cases.
